# Optimization of undersampling parameters for 3D intracranial compressed sensing MR angiography at 7 T

**DOI:** 10.1002/mrm.29236

**Published:** 2022-03-28

**Authors:** Matthijs H. S. de Buck, Peter Jezzard, Aaron T. Hess

**Affiliations:** ^1^ Wellcome Centre for Integrative Neuroimaging, FMRIB Division, Nuffield Department of Clinical Neurosciences University of Oxford Oxford UK

**Keywords:** MR angiography, time‐of‐flight MRA, compressed sensing, undersampling, lenticulostriate arteries, ultra‐high field

## Abstract

**Purpose:**

3D time‐of‐flight MRA can accurately visualize the intracranial vasculature but is limited by long acquisition times. Compressed sensing reconstruction can be used to substantially accelerate acquisitions. The quality of those reconstructions depends on the undersampling patterns used. In this work, we optimize sets of undersampling parameters for various acceleration factors of Cartesian 3D time‐of‐flight MRA.

**Methods:**

Fully sampled datasets, acquired at 7 Tesla, were retrospectively undersampled using variable‐density Poisson disk sampling with various autocalibration region sizes, polynomial orders, and acceleration factors. The accuracy of reconstructions from the different undersampled datasets was assessed using the vessel‐masked structural similarity index. Identified optimal undersampling parameters were then evaluated in additional prospectively undersampled datasets. Compressed sensing reconstruction parameters were chosen based on a preliminary reconstruction parameter optimization.

**Results:**

For all acceleration factors, using a fully sampled calibration area of 12 × 12 *k*‐space lines and a polynomial order of 2 resulted in the highest image quality. The importance of parameter optimization of the sampling was found to increase for higher acceleration factors. The results were consistent across resolutions and regions of interest with vessels of varying sizes and tortuosity. The number of visible small vessels increased by 7.0% and 14.2% when compared to standard parameters for acceleration factors of 7.2 and 15, respectively.

**Conclusion:**

The image quality of compressed sensing time‐of‐flight MRA can be improved by appropriate choice of undersampling parameters. The optimized sets of parameters are independent of the acceleration factor and enable a larger number of vessels to be visualized.

## 1 INTRODUCTION

Time‐of‐flight (TOF) MRA is a valuable technique for clinical study of the intracranial vasculature. It visualizes the blood in a certain region (slice or slab) by generating bright‐blood contrast between inflowing and stationary spins. In the brain, it can be used for detection of various types of vascular complications, such as atherosclerosis and stenosis^1^ or aneurysms.^2^, ^3^ Compared to other angiography techniques, such as CT angiography, TOF‐MRA has the benefit of being a noninvasive technique without the need for intravenous contrast agents and without exposing subjects to ionizing radiation.

High spatial resolution MRA can visualize small and highly tortuous vessels such as the lenticulostriate arteries (LSAs),^4^ which are implicated in up to a third of symptomatic strokes.^5^ MRA can be improved by using ultrahigh field MRI, with static magnetic field strengths of ≥ 7 Tesla (T).^6^ TOF‐MRA at 7 T benefits from longer T_1_ relaxation times and increased SNR, resulting in the potential for higher resolution acquisitions and improved visibility of small vessels.^4^ However, the achieved spatial resolution is limited by long acquisition times, which can lead to patient discomfort, increased patient movement, and increased clinical costs. In order to remain within clinical scan durations, sub‐Nyquist sampling techniques are required, such as parallel imaging techniques.^7^

Compressed sensing (CS)^8^ techniques have the potential to achieve high acceleration factors. CS combines highly undersampled nonuniform acquisitions and sparsity in a given domain to restore image quality. Due to the intrinsic sparsity of TOF‐MRA data in both the image and wavelet domain, and the improved MRA contrast at 7 T, it has already been shown that acceleration factors of 7.2^9^ or higher^10^, ^11^ can be achieved with a minimal reduction in clinical image quality^12^ or even with improved diagnostic image quality compared to conventional acceleration methods.^11^

Cartesian undersampled k‐space trajectories for 3D TOF‐MRA with CS reconstruction are commonly designed using 2D undersampling covering the 2 phase‐encode directions (k_*y*_, k_*z*_), with each sampled point in the (k_*y*_, k_*z*_) plane representing a continuously sampled line in the frequency‐encode direction (k_*x*_). Such undersampling masks are often created using pseudorandom variable‐density Poisson disks^13^ with a fully sampled calibration region in the center of the (k_*y*_, k_*z*_) plane.^9^, ^12^, ^13^, ^14^, ^15^, ^16^, ^17^ Variable‐density Poisson disk undersampling distributions are characterized by 3 parameters: (1) the undersampling factor (*R*), (2) the polynomial order of the sampling density variation (*pp*), and (3) the size of the fully sampled calibration region (*calib*).

Although the image quality depends on those undersampling parameters, no conclusive information is available about their optimal values for 3D TOF‐MRA at 7 T. It also remains unclear how the optimal acquisition parameters depend on the acceleration factor and resolution being used. Earlier work on reconstruction optimization for 2D‐multislice Nesterov reconstruction of 3 T TOF‐MRA found the best reconstruction results using the smallest of 3 calibration region sizes,^17^ but this smallest region for 2D MRA was still substantially larger than the calibration regions recently used for 7 T CS TOF‐MRA in 3D.^9^ Other work has compared the image quality from retrospectively undersampled 3D‐MRA data for various accelerations factors^10^, ^14^ or for the combination of acceleration factor and calibration region size in undersampled 2D MRI acquisitions for different contrasts.^18^ For dynamic MRI^19^ and numerical T_1_‐weighted brain models,^20^ studies into the optimization of undersampling parameters are available. However, it is unclear how this translates to the case of 3D TOF‐MRA, which requires the visibility of smaller structures in the reconstructed images and has the potential for higher acceleration factors due to the higher intrinsic sparsity.

In this work, which is an extension of Ref. 21, 3D TOF‐MRA undersampling parameters were optimized by retrospectively evaluating different calibration region sizes and polynomial orders at 6 acceleration factors (5 to 15). Finally, the identified undersampling patterns were evaluated in healthy subjects using additional prospectively undersampled acquisitions that adopted the optimized parameters.

## 2 METHODS

### 2.1 Data acquisition

Data were acquired using a 3D gradient‐echo noncontrast‐enhanced TOF‐MRA sequence on a Siemens (Erlangen, Germany) Magnetom 7 T scanner using a 1T×x32Rx head‐coil. All experiments were performed under an institutional agreement for technical development in accordance with International Electrotechnical Commission and UK Health Protection Agency guidelines. In each subject, 4 sequential slabs^22^ consisting of 640 × 506 × 56 voxels with a resolution of (0.31 mm)^3^ were acquired using a slab overlap of 19.64% and a combined FOV of 200 × 157 × 60 mm^3^. Further sequence parameters were: TR/TE = 14/5.61 ms (allowing for asymmetric echo), flip angle = 20°, and bandwidth = 118 Hz/pixel. To reduce SAR, an increased excitation pulse duration of 1.536 ms was used. No motion correction was used. The acquisition time was 26:39 min for a fully sampled acquisition.

Fully sampled datasets from 2 subjects were used for parameter optimization through retrospective undersampling (Cohort 1). Additional datasets were acquired using a modified TOF‐MRA sequence that utilized predefined variable‐density Poisson disk undersampling masks. A fully sampled acquisition and 4 different undersampled datasets, using both the optimized and more conventional undersampling parameters, were acquired from 7 healthy volunteers (Cohort 2). The reconstructed image quality for those additional datasets was compared to assess consistency of the findings.

### 2.2 Undersampling

Undersampling masks were generated using the SPIRiT Toolbox v0.3,* characterized by the 3 previously described parameters (R,pp,andcalib). A polynomial order of 0 corresponds to homogeneous undersampling, with higher values corresponding to denser sampling (i.e., lower Poisson disk radii) closer to the center of k‐space. Larger calibration regions (consisting of calib×calib continuously sampled frequency‐encode lines) improve the estimation of coil sensitivities^23^ but take up more scan time, thereby reducing the available time to acquire other regions of k‐space for scan time‐matched acquisitions. Undersampling was performed retrospectively for the fully sampled data acquired in Cohort 1 at acceleration factors ranging from 5 to 15. The calibration region size was chosen to range from the lower limit for the ESPIRiT tool (*calib* = 10) to the highest value found in the literature for 3D TOF‐MRA.^9^ In Cohort 2, prospectively undersampled data were acquired for validation at *R* = 7.2 (which was previously found to be “a reasonable trade‐off between scan time and image quality”^9^, p. 201) and *R* = 15. Note that the undersampling masks in this work were generated using the SPIRiT Toolbox, which uses a polynomial variation for the sampling density distribution (given by the value of *pp*). Alternative ways of varying the sampling density distribution can also be used for Poisson disk undersampling, such as the Gaussian density distribution used in Ref. 9.

### 2.3 Reconstruction

For all reconstructions, coil sensitivities were estimated using ESPIRiT^23^ based on the fully sampled calibration region in the center of k‐space. For the reconstruction of undersampled data, compressed sensing was implemented using a Fast Iterative Shrinkage‐Thresholding Algorithm (FISTA)^24^ with ℓ1‐regularization in the wavelet domain^17^, ^25^ using the pics (parallel imaging and compressed sensing) tool in the Berkeley Advanced Reconstruction Toolbox^26^, ^27^ (BART; v0.4.02). The ℓ1‐regularization seeks to find the solution to:

(1) $$\min_{x}\left\{\left|A\left(x\right)-b\right|+\lambda {\left|\phi \left(x\right)\right|}_{1}\right\},$$

where **b** represents the acquired (undersampled) k‐space data; **A** is the undersampled Fourier Transform operator over the reconstructed image **x**; λ is a regularization parameter; and ϕ denotes the Daubechies wavelet transform. The first term (|A(x)−b|) ensures data consistency, whereas the second term ($\lambda {\left|\phi \left(x\right)\right|}_{1}$) enforces sparsity in the wavelet domain.

Before the undersampling parameter optimization was performed, appropriate values for λ and the number of iterations (*n*_iter_) were first established by reconstructing 2 different undersampled datasets (at R=9 and R=13) for each subject using various values for *n*_iter_ and λ, and comparing the resulting reconstructions to the fully sampled reference data. Parameter values ($\lambda =0.007,n_{\text{iter}}=20$) that consistently returned good reconstruction results were used for all other reconstructions; see Supporting Information Figure S1. The initial reconstruction parameter optimization was performed using arbitrarily chosen undersampling masks. For validation, it was repeated at the end of the study using the established optimized undersampling masks to ensure that the initial optimized reconstruction parameters were still consistent.

Reconstructions were performed offline using an Intel (Intel, Santa Clara, CA) Xeon CPU E5‐2680v4 running at 2.40 GHz with 14 cores and 28 logical processors. A single‐slab CS reconstruction took approximately 14 min.

### 2.4 Quantification of reconstruction quality

Previous work found that when comparing reconstructions from retrospectively undersampled TOF‐MRA data to the corresponding fully sampled data, the vessel‐masked structural similarity index (SSIM) correlates very well with visual evaluation by radiologists.^28^ The root‐mean‐square error, which is also often used in the literature, was found in Ref. 28 to result in low agreement with visual evaluation by radiologists and is therefore not used in this work.

**FIGURE 1:**
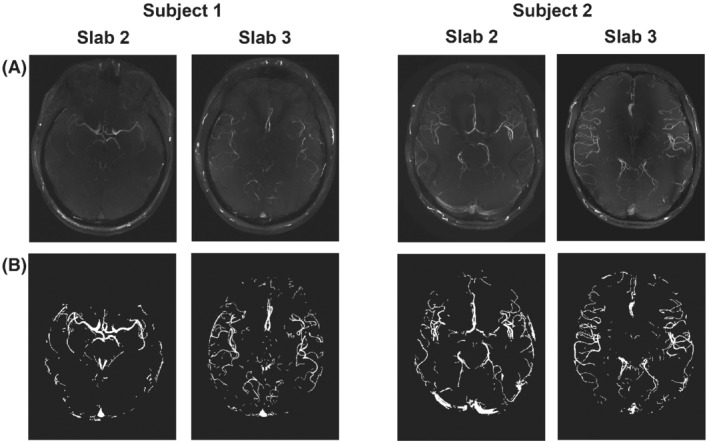
The 4 slabs used to test retrospective undersampling strategies (from Cohort 1). (A) MIPs of the fully sampled reconstruction for each slab. (B) The corresponding vessel masks, which were used for computing the vessel‐masked SSIM^27^. MIP, maximum intensity projection; SSIM, structural similarity index

The structural similarity index is a quantitative estimate of the perceived visual agreement between a set of images based on local intensity variations. Higher SSIM indicates greater agreement between a set of images, with a value of 1 indicating perfect agreement. Here, the SSIM is calculated for maximum intensity projections (MIPs) normalized to the 99th intensity percentile. The mean SSIM is calculated over a vessel‐masked region (Figure 1B), which reduces the sensitivity to variations in the background signal.

The SSIM requires spatial consistency of the information in the 2 images being compared. This limits its use when comparing prospectively undersampled datasets because minor subject motion between consecutive scans significantly influences the SSIM. Therefore, a metric (‘number of detected peaks’) to estimate the change in the number of visible vessels in an image^9^ was used to assess the quality of prospectively undersampled data. From MIPs, 100 cross‐sectional lines in the left–right direction were taken, covering the central 50% of the FOV in the anteroposterior direction. Along these lines, peaks in the intensity profiles were detected using the findpeaks function in MatLab R2019a (MathWorks, Natick, MA) with a minimum peak prominence of 0.15.

To quantify the difference in visibility of small vessels in prospectively undersampled acquisitions, a comparable approach was used to estimate the visibility of the LSAs in coronal projections.

## 3 RESULTS

### 3.1 Fully sampled data

Figure 1 shows MIPs of the fully sampled reconstructions of the central 2 (out of 4) slabs for both subjects in Cohort 1, which were used for parameter optimization. Those slabs were used as the ground‐truth reference for all combinations of parameters used to assess the different retrospectively undersampled reconstructions. Only 2 slabs per subject were used to remain within a reasonable computation time. The central slabs were found to contain vessels of various sizes and degrees of tortuosity (Figure 1), making it possible to compare undersampling parameter optimization results for different parts of the vasculature.

### 3.2 Undersampling optimization

**FIGURE 2:**
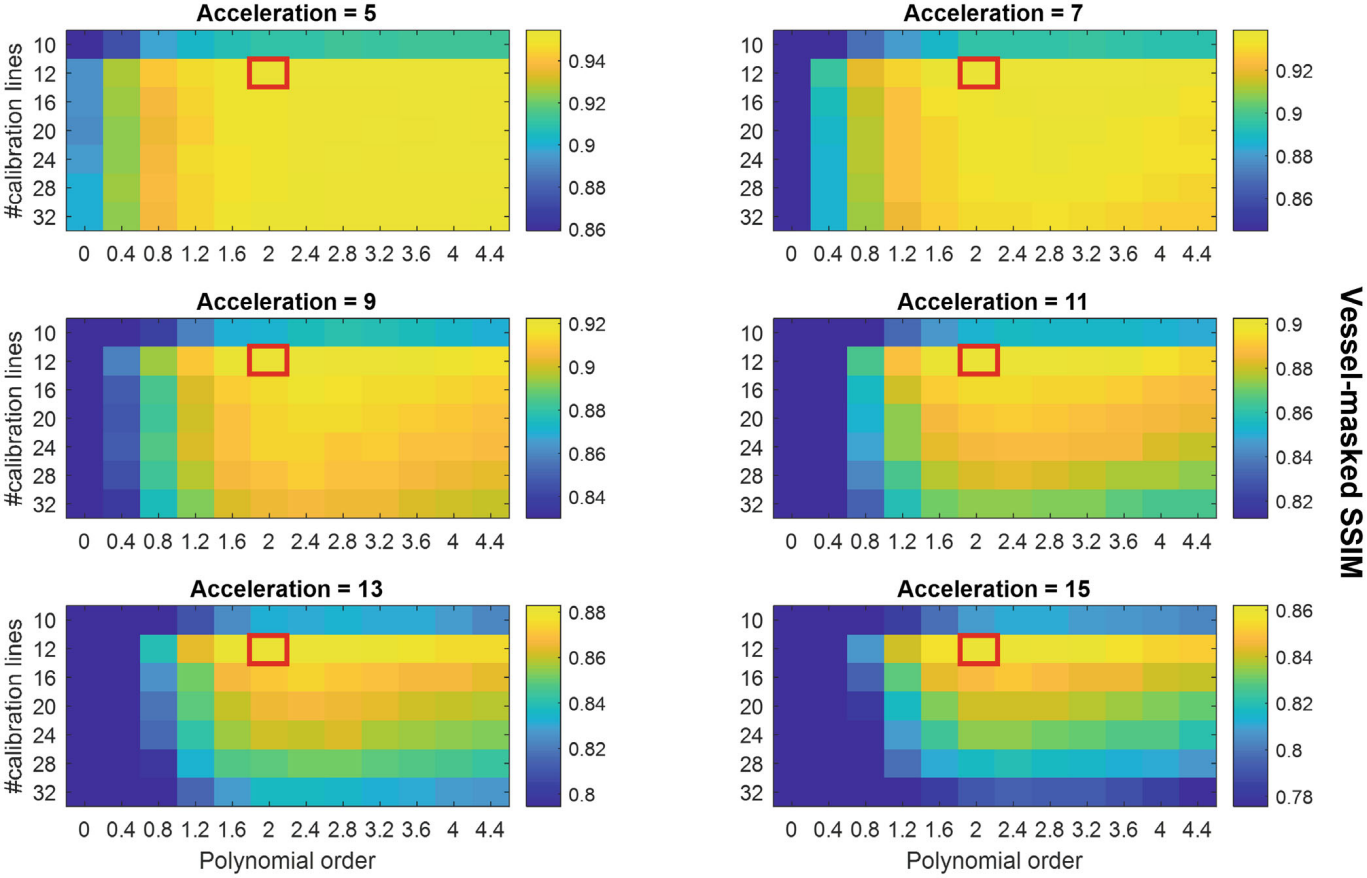
Average SSIMs for various sets of undersampling parameters. Each datapoint represents the mean vessel‐masked SSIM values of the 4 slabs shown in Figure 1. The results for each of the 4 slabs are separately shown in Supporting Information Figures S3‐S6. The scaling of the SSIM values in each individual figure runs from the maximum value (for the given acceleration factor) to 90% of that maximum value to maximize the visibility of the relative image quality for each acceleration factor. Red boxes indicate the proposed optimized undersampling parameters

The mean vessel‐masked SSIMs resulting from the retrospective undersampling parameter optimization for 6 acceleration factors *R* (5 to 15) for all slabs (in Figure 1) are presented in Figure 2. Each of the values in Figure 2 is the mean value for 2 different undersampling masks (using the same undersampling parameters) such that each datapoint is the average of 8 comparisons: 4 different slabs with 2 different undersampling masks each. Supporting Information Figures S3–S6 show the results for each individual slab.

Especially for higher acceleration factors, Figure 2 indicates that for matched scan time an optimal set of undersampling parameters occurs for 12 × 12 calibration lines (‘*calib* = 12’) with a polynomial order of approximately 2.0 to 2.4. For datasets reconstructed from the Cohort 1 data with a downsampled spatial resolution (0.5 mm and 0.6 mm isotropic), optimal undersampling parameters consisted of 12 × 12 calibration lines and a polynomial order of approximately 1.6 to 2.0 (data not shown).

For validation, the initial reconstruction parameter optimization was repeated for those optimized undersampling parameter values; see Supporting Information Figure S2, which confirms generally good performance of the previously established reconstruction parameter values ($\lambda =0.007,n_{\text{iter}}=20$).

**FIGURE 3:**
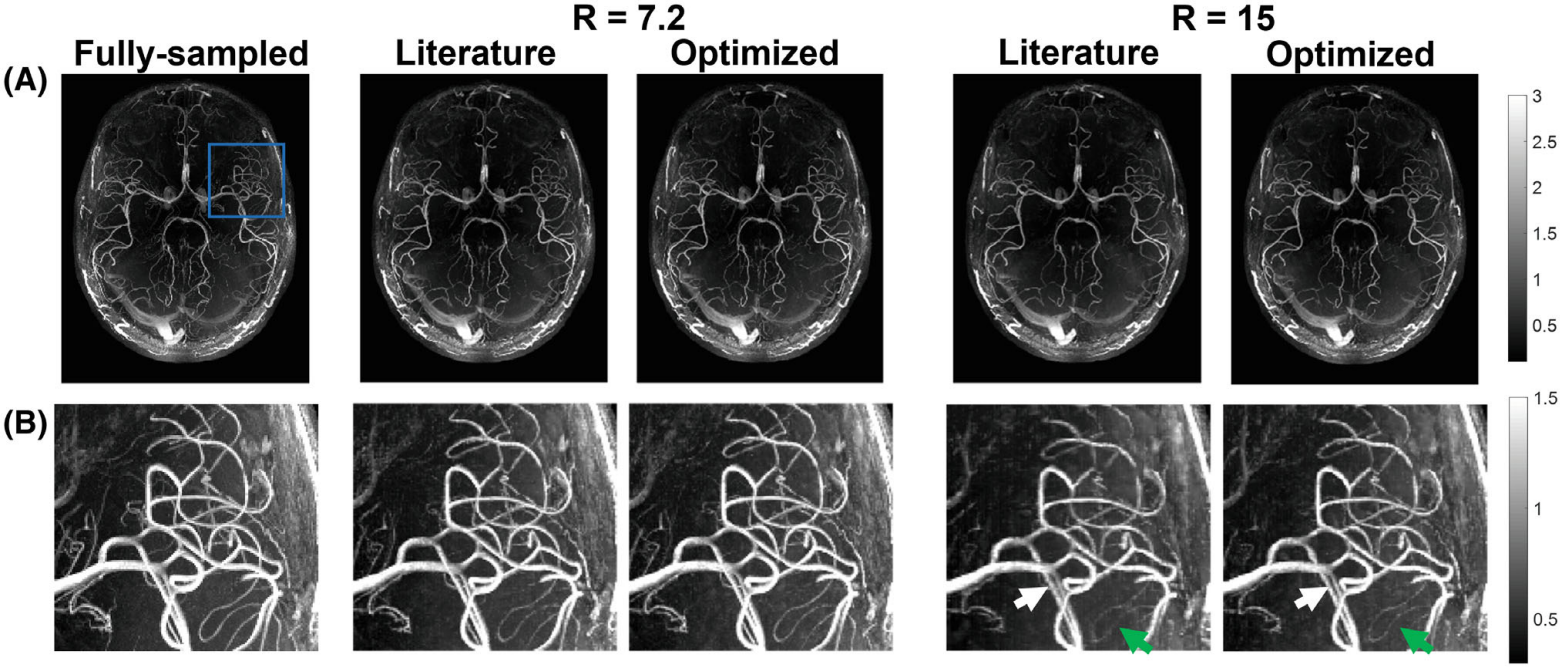
Comparison of axial MIPs from optimized (*calib* = 12) and literature‐based (*calib* = 32) undersampling schemes for *R* = 7.2 and *R* = 15. (A) Reconstructed images from fully sampled data and the different prospectively undersampled acquisitions. (B) Closeup of the region marked with a blue square in (A) for all acquisitions. Green arrows indicate examples of improved vessel visibility when using optimized undersampling parameters; white arrows indicate improved vessel sharpness. The windowing was reduced for (B) to improve the visibility of small vessels. *calib*, calibration region size; *R*, undersampling factor

**FIGURE 4:**
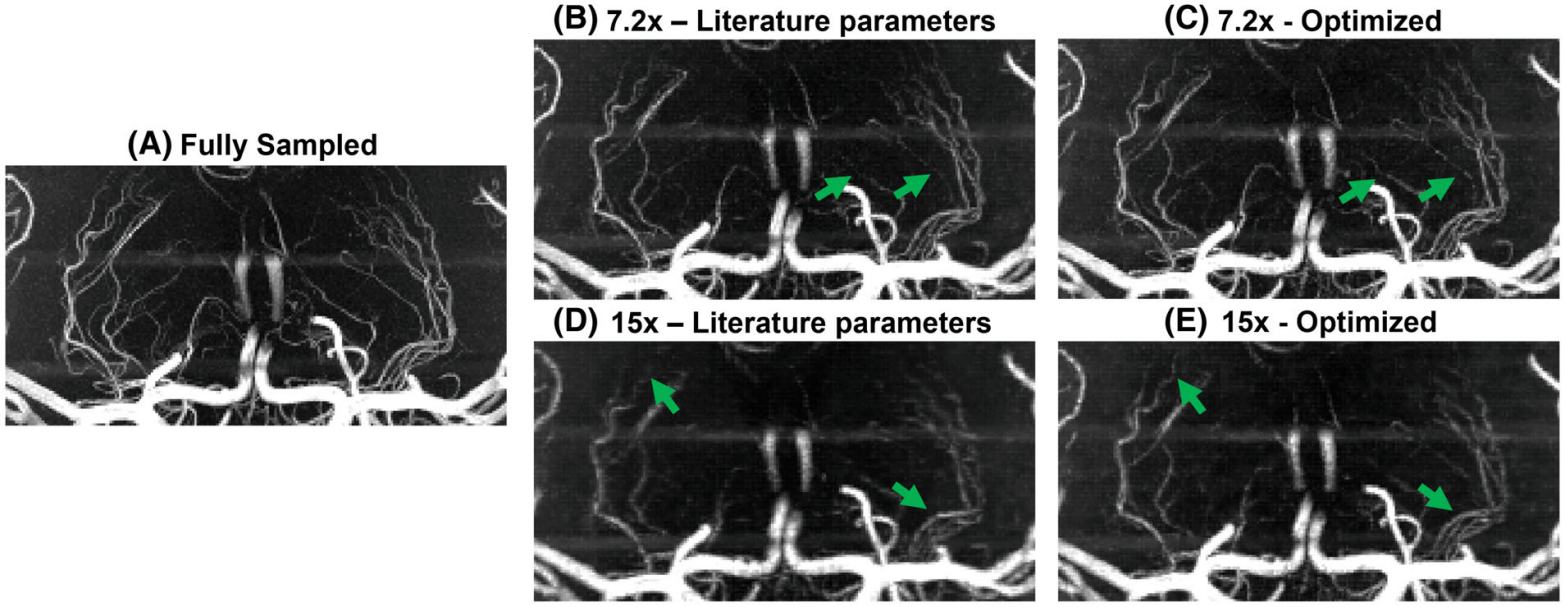
Comparison of coronal MIPs of the LSAs from optimized and literature‐based undersampling schemes. Images shown for (A) fully sampled data; (B,C) data for *R* = 7.2 using literature‐based (B) and optimized (C) prospectively undersampled acquisitions; and (D,E) data for *R* = 15 using literature‐based (D) and optimized (E) prospectively undersampled acquisitions. LSA, lenticulostriate arteries

Figures 3 and 4 show example reconstructions from fully sampled data, prospectively undersampled data with “literature‐based” parameters,^9^ and prospectively undersampled data using “optimized” parameters (Cohort 2). In these figures optimized undersampling masks were generated using calib=12 and pp=2.0, and literature‐based masks were generated using calib=32 and pp=2.0 with matched scan time; see Supporting Information Figure S7.

**FIGURE 5:**
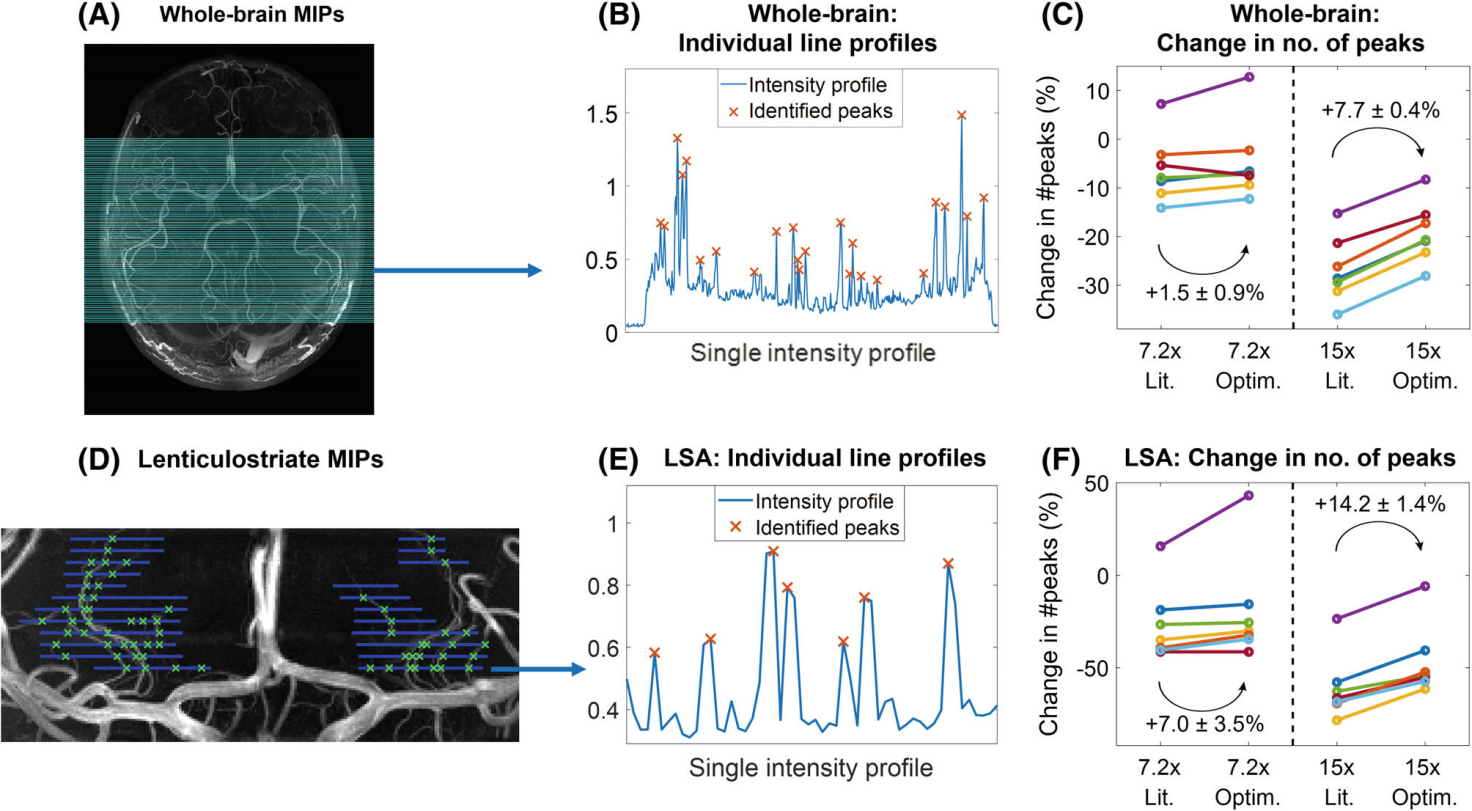
Quantification of the change in the number of detected peaks. (A–C) Whole‐brain MIPs. (A) The 100 lines along which peaks in the intensity profiles were identified on all datasets (as shown in (B) for line 25). (C) The change in the identified number of peaks for optimized and literature‐based undersampled acquisitions, relative to fully sampled acquisitions. Each color indicates a subject from Cohort 2. (D–F): The same as (A–C) for MIPs of lenticulostriate ROIs. Line locations were drawn manually at 5‐pixel intervals. Lit., literature‐based; Optim., optimized; ROI, region of interest

Figure 5 shows examples of the implementation of the ‘number of detected peaks’‐metric, as well as the resulting differences between the various undersampling schemes for the 7 volunteers in Cohort 2. Results are shown for whole‐brain axial MIPs and coronal MIPs of the lenticulostriate region.

At *R* = 7.2, the mean relative number of detected peaks when using 12 × 12 compared to 32 × 32 calibration lines increases by 1.5 ± 0.9 percentage points (from −6.1% to −4.6%) for axial MIPs and by 7.0 ± 3.5 percentage points (−26.5% to −19.5%) for the LSAs. At *R* = 15, the number of detected peaks increases by 7.7 ± 0.4 percentage points (−26.9% to −19.2%) for axial MIPs and by 14.2 ± 1.4 percentage points (−60.9% to −46.7%) for the LSAs.

The change in the number of detected peaks when using optimized undersampling parameters is statistically significant at *R* = 15, based on 1‐sided paired‐sample *t* tests: *p* < 0.001 for both whole‐brain and LSA MIPs and at *R* = 7.2 for LSA MIPs (*p* = 0.047). At *R* = 7.2, statistical significance was not reached for the whole‐brain MIPs, but a trend value of *p = 0.063* was obtained.

## 4 DISCUSSION

Although differences in the image quality when using various autocalibration region sizes and polynomial orders are visible for all acceleration factors in Figure 2, the relative importance of using optimized parameters increases at higher acceleration factors. For all acceleration factors, 12 × 12 calibration lines with a polynomial order of 2 to 2.4 consistently yielded the best reconstruction accuracy. Simulation results at reduced spatial resolutions indicate the same optimal calibration region size but with slightly lower polynomial orders (approximately 1.6 to 2.0 at 0.6 mm isotropic). For all resolutions, optimization of the calibration region size has a bigger influence on the image quality than optimization of the polynomial order. This optimal calibration region size is considerably smaller than values found in the literature. Reducing the calibration region size from 32 × 32 to 12 × 12 lines in k‐space corresponds to an 86% reduction in the amount of scan time required for scanning this central k‐space region. For a fixed scan time, this makes it possible to spend more scan time acquiring data at higher spatial frequencies, explaining the observed improvement in small vessel visibility. Asymmetric calibration regions, with reduced coverage in the partition direction (k_*z*_), were not included in this work because of limitations in the used implementation of ESPIRiT.

For the prospectively undersampled data, the performance of these optimized undersampling parameter values is compared to those taken from Ref.^9^. Figures 3 and 4 show a clear reduction in the number of visible vessels at high acceleration factors using both optimized and literature‐based acquisition parameters compared to a fully sampled acquisition. However, vessel visibility and sharpness noticeably improve when using optimized undersampling parameters versus literature parameters, especially for small vessels and at high acceleration factors. Although accurate quantitative comparison of different prospectively undersampled acquisitions using SSIM is not possible due to subject motion between scans, the reduction in signal loss when using optimized parameters was quantitatively approximated using the detection of vascular signal peaks. The identified peak locations in reconstructions from prospectively undersampled data are consistent with the locations of the peaks in the corresponding fully sampled data (not shown), indicating that the metric gives a representative approximation of relative image quality.

Prospectively and retrospectively undersampled data can differ in image quality because of the possibility of eddy‐current artifacts due to larger gradient switching in prospectively undersampled acquisitions and different amounts of total subject motion due to the different scan times. However, the observed improvement in image quality when using optimized undersampling schemes identified using the retrospectively undersampled data (Figure 2) is consistent with the results found in Figures 3, 4, 5 for prospectively undersampled data. This applies to both the improvement in image quality when using smaller calibration regions (in particular, 12 × 12 calibration lines) and to the increased importance of acquisition parameter optimization at higher acceleration factors. This improvement when using optimized undersampling parameters can be achieved without increasing scan time or reconstruction time, and without additional technical requirements. This improvement in image quality at a given acceleration can also be interpreted as an opportunity to increase acceleration factor to achieve equal image quality (Supporting Information Figure S8). For example, a scan duration of 2:36 min (*R* = 11) when using 32 × 32 calibration lines provides comparable results to a scan duration of 1:57 min (*R* = 15) using 12 × 12 calibration lines.

Because a small calibration region size was observed to improve performance, we also evaluated reconstructions from k‐space data without calibration regions using simultaneous autocalibration and k‐space estimation (SAKE)^29^ for sensitivity estimation (Supporting Information Figure S9). This did not provide improved image quality compared to the reconstructions using 12 × 12 sampled lines in k‐space.

For 1 of the 7 volunteers in Cohort 2, an increase in the number of detected peaks is visible in the undersampled acquisitions (purple data in Figure 5). This is likely caused by a reduction in image quality in the fully sampled dataset due to subject motion during that scan, highlighting a benefit of accelerated acquisitions.

The data used in this work were acquired using a relatively simple protocol, which does not make use of techniques such as additional (e.g., fat or venous) signal saturation,^9^, ^30^, ^31^ parallel transmission,^32^, ^33^ variable‐rate selective excitation (VERSE)‐shimming,^9^, ^30^, ^34^ or intravenous contrast agents.^35^, ^36^ Such techniques can enhance contrast and increase sparsity and thereby improve the CS reconstruction results. However, the optimal undersampling parameters were found to be consistent for volumes with high differences in vascular characteristics and visibility (Figure 2) and are therefore also expected to remain consistent for different contrasts. The improvement in image quality when using 12 × 12 calibration lines was also found to be consistent for data acquired using various acceleration factors and spatial resolutions. Although 32‐channel receive coils are most commonly used in 7 T MRI, it remains unclear how the results presented here would translate to different coil configurations.

Previous work on the optimization of acquisition parameters for CS T_1_‐weighted MRI in 3D found that optimized sampling schemes require increasingly dense sampling in the center of k‐space for higher acceleration factors,^20^ and that the extent of the calibration region should be as high as possible for 2D‐MRI.^18^ This is different from the results found here, with an optimized set of acquisition parameters that appears to be consistent for all acceleration factors and that uses a small calibration region. This difference may be explained by the inherently sparser image contrast of MRA compared to T_1_‐weighted MRI and the use of 3D instead of 2D k‐space data. Because this sparser image signal is contained in high‐frequency areas of k‐space, sampling at off‐center locations of k‐space remains important at higher acceleration factors for MRA.

## 5 CONCLUSION

Optimized undersampling parameters for 3D MRA at 7 T using compressed sensing reconstruction were established. For all acceleration factors, the highest image quality was achieved by using a fully sampled calibration area of 12 × 12 lines and a polynomial order of 2. Although the optimized undersampling parameters were the same for all acceleration factors, the importance of using optimized undersampling parameters was found to increase for higher acceleration factors.

## CONFLICT OF INTEREST

Matthijs de Buck receives studentship support from Siemens Healthineers. Peter Jezzard is the Editor‐in‐Chief of *Magnetic Resonance in Medicine*. In line with COPE guidelines, he wishes to recuse himself from all involvement in the review process of this paper, which was handled by an associate editor. He and the other authors have no access to the identity of the reviewers.

## Supporting information


**FIGURE S1.** Results of the initial reconstruction parameter optimization, used to estimate appropriate values for the number of iterations and the regularization parameter *λ*. Reconstruction accuracy is given using **(a)** the mean vessel‐masked SSIM and **(b)** the number of detected peaks. Results are shown for two different imaging volumes, each undersampled using two different sets of undersampling parameters (as indicated at the top of both columns). Red boxes indicate the set of reconstruction parameters (*λ* = 0.007, 20 iterations) used for all later reconstructions because of the consistently good results using both metrics, within reasonable reconstruction times.
**FIGURE S2.** The results presented in Supporting Information Figure S1, calculated using the “optimized” undersampling masks (as indicated at the top of both columns) used for retrospective undersampling at a later stage in this study. The parameter combinations (*λ* = 0.007, 20 iterations) indicated by the red boxes still provide consistently good results reasonable reconstruction times.
**FIGURE S3.** The vessel‐masked SSIM‐values comparing the fully sampled reference datasets and corresponding reconstructed datasets for Subject 1, Slab 2.
**FIGURE S4.** The vessel‐masked SSIM‐values comparing the fully sampled reference datasets and corresponding reconstructed datasets for Subject 1, Slab 3.
**FIGURE S5.** The vessel‐masked SSIM‐values comparing the fully sampled reference datasets and corresponding reconstructed datasets for Subject 2, Slab 2.
**FIGURE S6.** The vessel‐masked SSIM‐values comparing the fully sampled reference datasets and corresponding reconstructed datasets for Subject 2, Slab 3.
**FIGURE S7.** The four undersampling masks used for the acquisition of prospectively undersampled data (Figures 3, 4, 5 in the main text). Masks (a) and (c) correspond to the “Literature‐based” undersampling parameters, while (b) and (d) are generated using the “Optimized” parameters, as specified in the figures. Since the total amount of acquired data is fixed for a certain acceleration factor, undersampling masks with smaller calibration region sizes contain more k‐space locations outside the calibration region.
**FIGURE S8.** Reconstructed image quality for different undersampling approaches, from retrospectively undersampled data. Results are shown using **(a)** the vessel‐masked SSIM and **(b)** the error in the number of detected peaks. Black arrows indicate the increase in acceleration factor when using calib = 12 (at R = 15) instead of 32, which can be achieved without loss in image quality.
**FIGURE S9.** Comparison of image quality in reconstructions from retrospectively undersampled k‐space data without calibration regions, using SAKE‐calibration for sensitivity estimation, to image quality in reconstructions using 12 × 12 sampled lines in k‐space for calibration. After sensitivity estimation, both approaches were reconstructed using the same compressed sensing pipeline. Results are shown as the mean SSIM ± the standard deviation across the 4 slabs shown in Figure 1.

## Data Availability

In support of *Magnetic Resonance in Medicine's* reproducible research goal, the MatLab (MathWorks) pipeline that was used for the retrospective undersampling parameter optimization is available on the Oxford Research Archive (https://doi.org/10.5287/bodleian:VJZVxnAOb). On request, the authors can also provide specific datasets used in this work.

## Note

*
https://people.eecs.berkeley.edu/∼mlustig/Software.html. Note that in this work the acceleration factor refers to the *effective* acceleration (i.e., including the time taken to scan the calibration region), which is different from the definition of the “acc” parameter in the SPIRiT‐vdPoisMex tool.
