# Supplementary material for: Optimization of undersampling parameters for 3D intracranial compressed sensing MR angiography at 7 T
Source: Magn Reson Med. 2022 Mar 28;88(2):880–9. doi: 10.1002/mrm.29236 (PMC9314035; doi:10.1002/mrm.29236)
Supplement: Supplementary file 1 — FIGURE S1. Results of the initial reconstruction parameter optimization, used to estimate appropriate values for the number of iterations and the regularization parameter λ. Reconstruction accuracy is given using (a) the mean vessel‐masked SSIM and (b) the number of detected peaks. Results are shown for two different imaging volumes, each undersampled using two different sets of undersampling parameters (as indicated at the top of both columns). Red boxes indicate the set of reconstruction parameters (λ = 0.007, 20 iterations) used for all later reconstructions because of the consistently good results using both metrics, within reasonable reconstruction times. FIGURE S2. The results presented in Supporting Information Figure S1, calculated using the “optimized” undersampling masks (as indicated at the top of both columns) used for retrospective undersampling at a later stage in this study. The parameter combinations (λ = 0.007, 20 iterations) indicated by the red boxes still provide consistently good results reasonable reconstruction times. FIGURE S3. The vessel‐masked SSIM‐values comparing the fully sampled reference datasets and corresponding reconstructed datasets for Subject 1, Slab 2. FIGURE S4. The vessel‐masked SSIM‐values comparing the fully sampled reference datasets and corresponding reconstructed datasets for Subject 1, Slab 3. FIGURE S5. The vessel‐masked SSIM‐values comparing the fully sampled reference datasets and corresponding reconstructed datasets for Subject 2, Slab 2. FIGURE S6. The vessel‐masked SSIM‐values comparing the fully sampled reference datasets and corresponding reconstructed datasets for Subject 2, Slab 3. FIGURE S7. The four undersampling masks used for the acquisition of prospectively undersampled data (Figures 3, 4, 5 in the main text). Masks (a) and (c) correspond to the “Literature‐based” undersampling parameters, while (b) and (d) are generated using the “Optimized” parameters, as specified in the figures. Since the total amount [file MRM-88-880-s001.docx]

**Supporting Information**


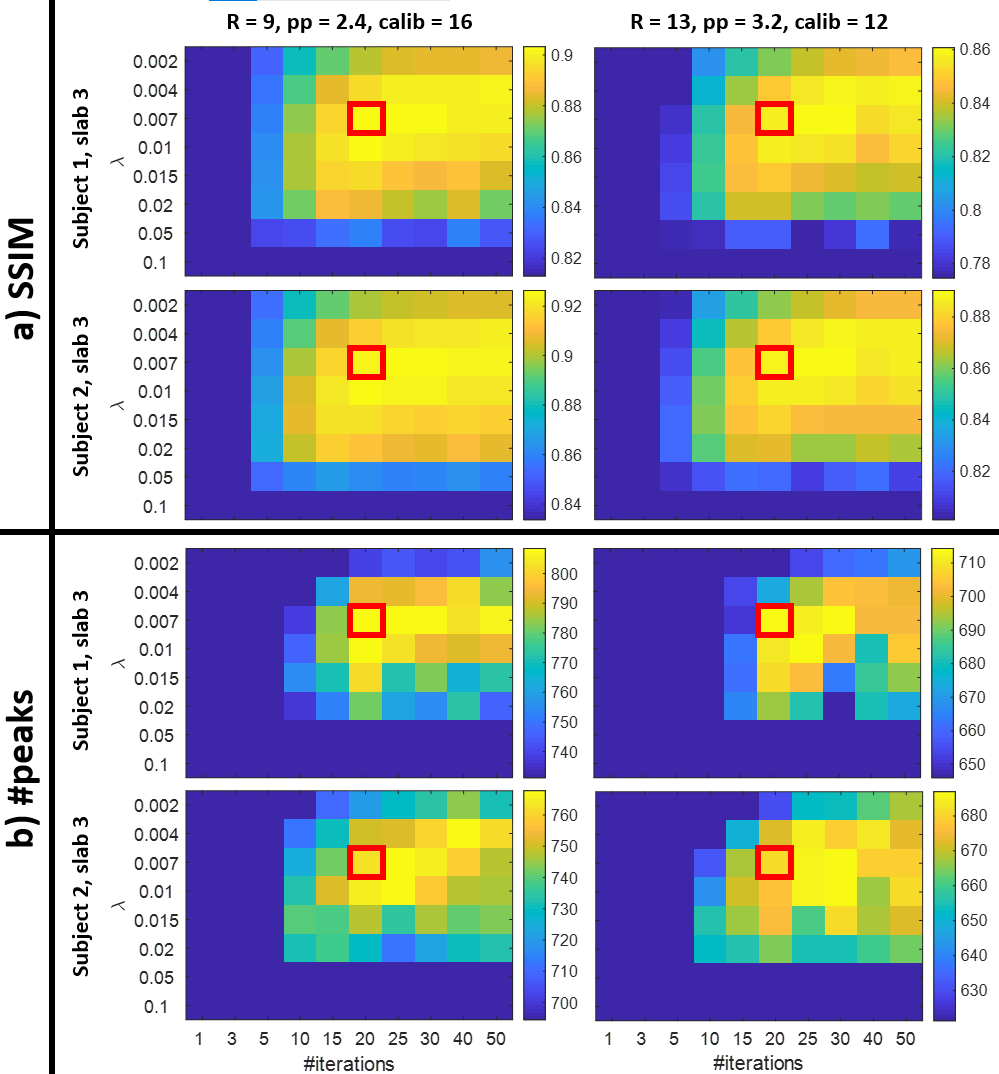


Supporting Information Figure S1: Results of the initial reconstruction parameter optimization, used to estimate appropriate values for n_iter_ and the regularization parameter $\boldsymbol{\lambda}$. Reconstruction accuracy is given using (a) the mean vessel-masked SSIM and (b) the number of detected peaks. Results are shown for two different imaging volumes, each undersampled using two different sets of undersampling parameters (as indicated at the top of both columns). Red boxes indicate the set of reconstruction parameters ($\boldsymbol{\lambda=0.007}$, 20 iterations) used for all later reconstructions because of the consistently good results using both metrics, within reasonable reconstruction times.


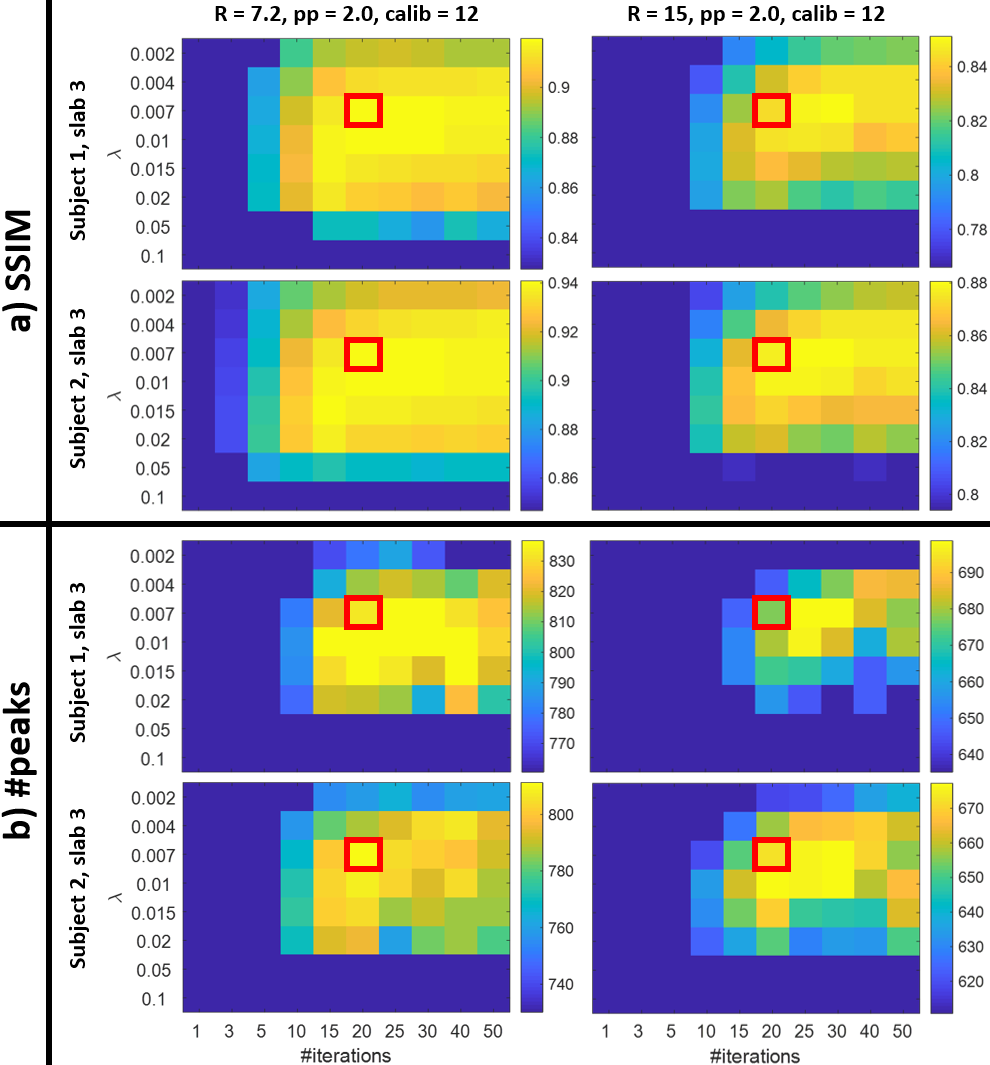


Supporting Information Figure S2: The results presented in Supporting Information Figure S1, calculated using the “optimized” undersampling masks (as indicated at the top of both columns) used for retrospective undersampling at a later stage in this study, using data acquired from Cohort 1. The parameter combinations ($\boldsymbol{\lambda=0.007}$, 20 iterations) indicated by the red boxes still provide consistently good results reasonable reconstruction times.


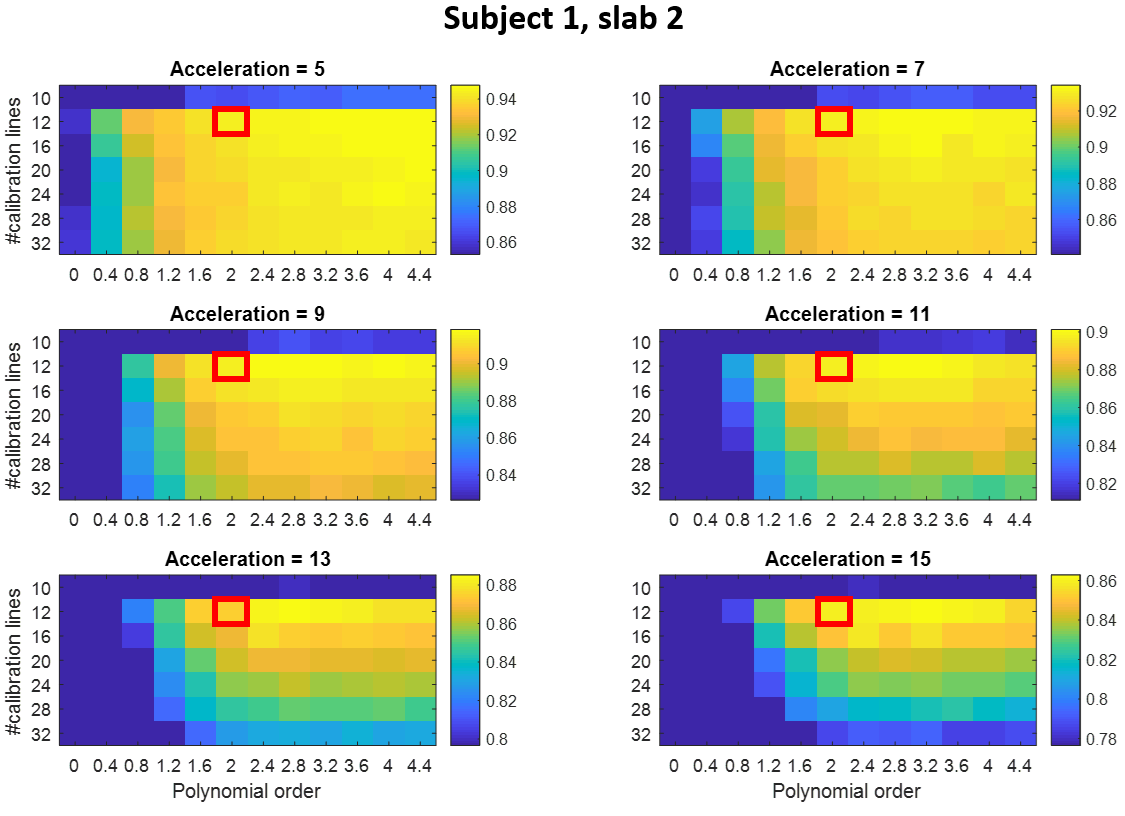


Supporting Information Figure S3: The vessel-masked SSIM-values comparing the fully sampled reference datasets and corresponding reconstructed datasets for Subject 1, Slab 2. Red boxes indicate the proposed optimized undersampling parameters.


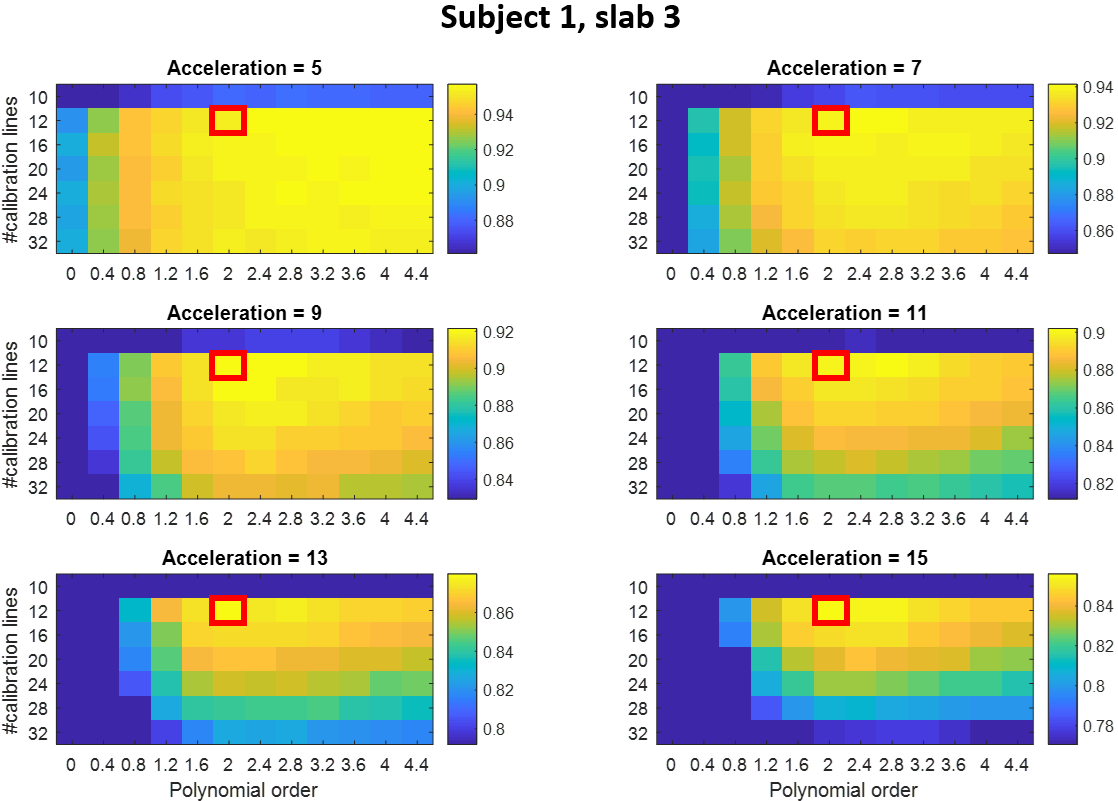


Supporting Information Figure S4: The vessel-masked SSIM-values comparing the fully sampled reference datasets and corresponding reconstructed datasets for Subject 1, Slab 3. Red boxes indicate the proposed optimized undersampling parameters.


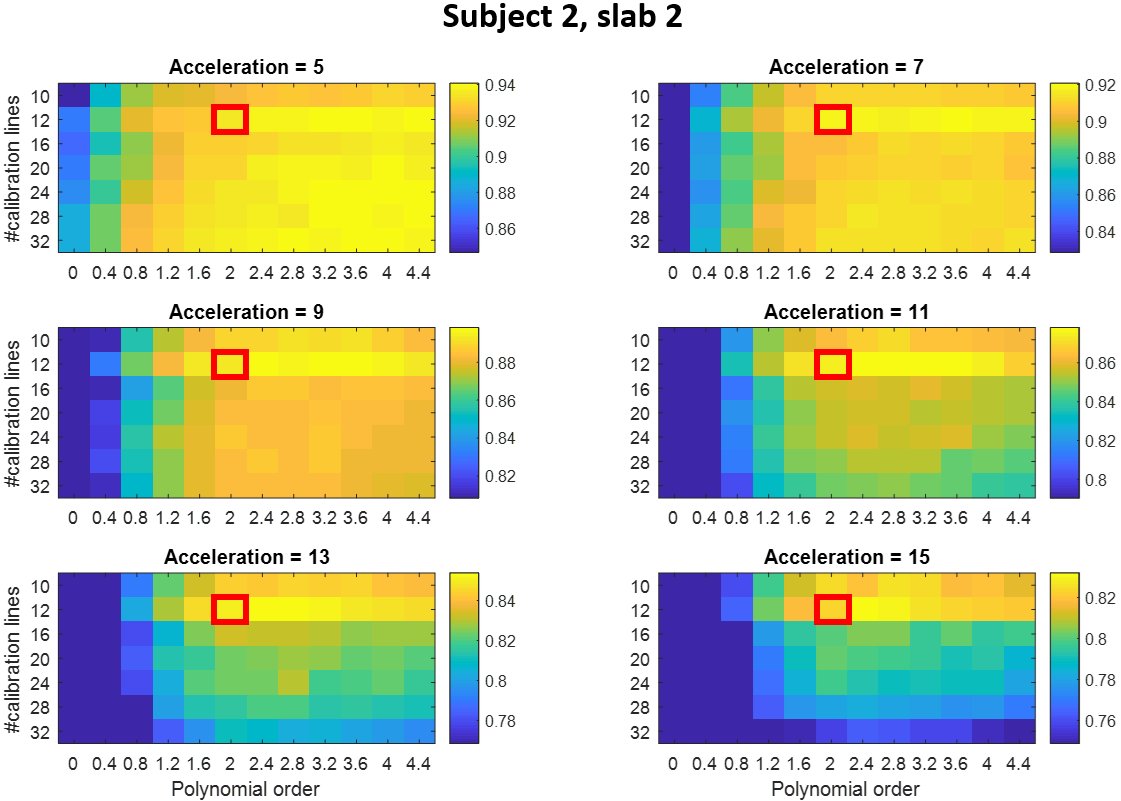


Supporting Information Figure S5: The vessel-masked SSIM-values comparing the fully sampled reference datasets and corresponding reconstructed datasets for Subject 2, Slab 2. Red boxes indicate the proposed optimized undersampling parameters.


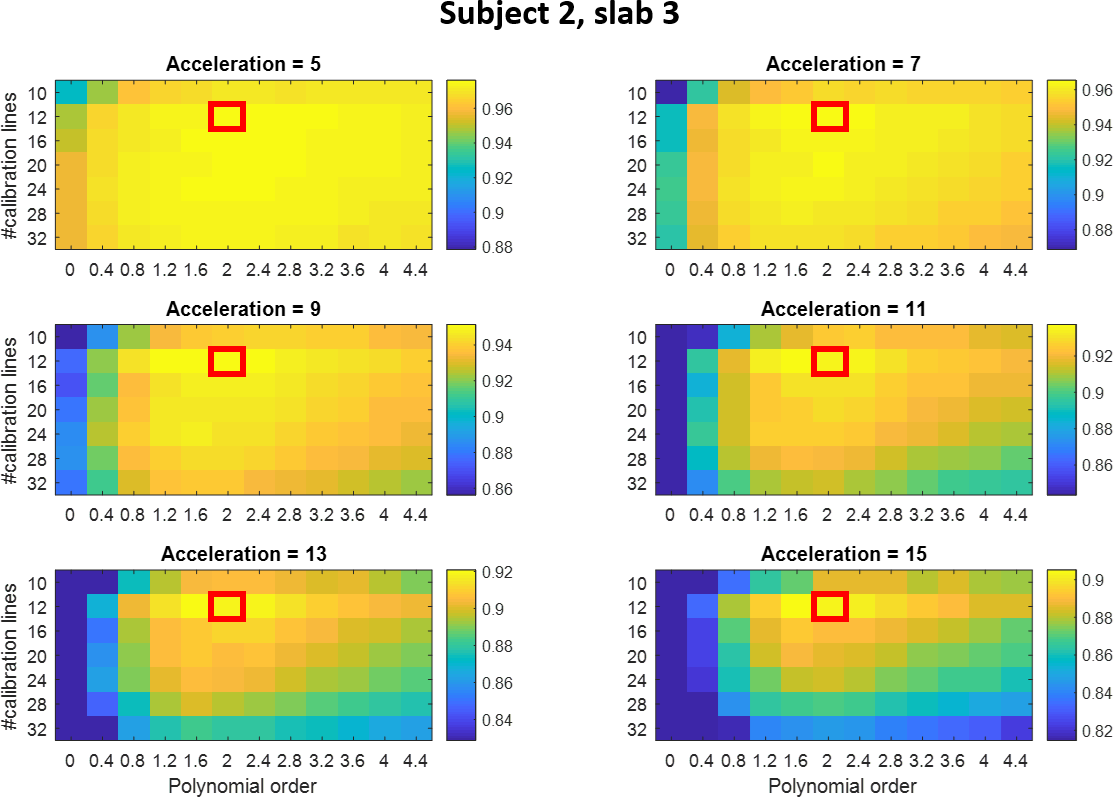


Supporting Information Figure S6: The vessel-masked SSIM-values comparing the fully sampled reference datasets and corresponding reconstructed datasets for Subject 2, Slab 3. Red boxes indicate the proposed optimized undersampling parameters.


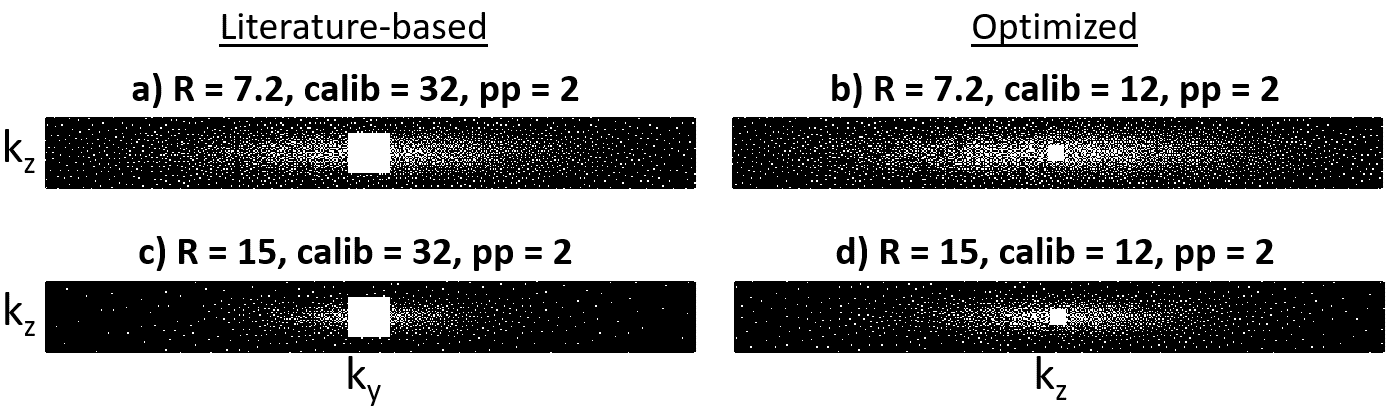


Supporting Information Figure S7: The four undersampling masks used for the acquisition of prospectively undersampled data (Figures 3-5 in the main text). Masks (a) and (c) correspond to the “Literature-based” undersampling parameters, while (b) and (d) are generated using the “Optimized” parameters, as specified in the figures. Since the total amount of acquired data is fixed for a certain acceleration factor, undersampling masks with smaller calibration region sizes contain more k-space locations outside the calibration region.


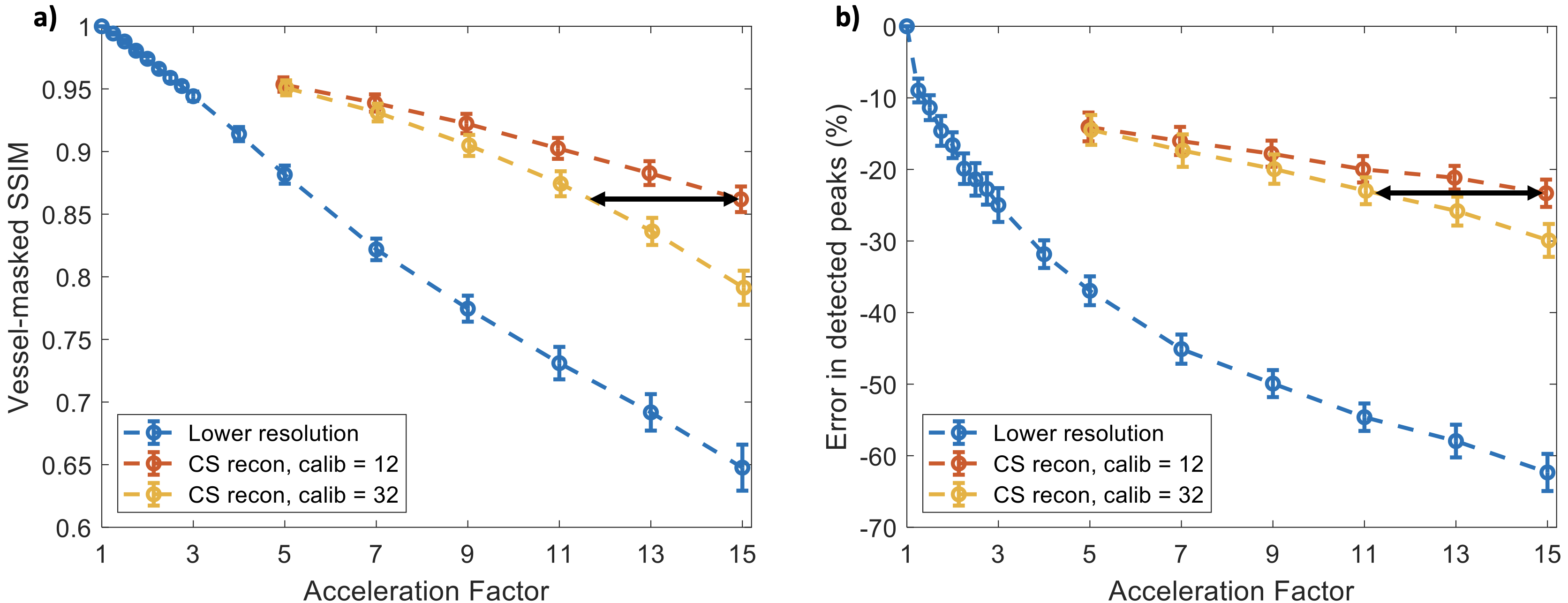


Supporting Information Figure S8: Reconstructed image quality for different undersampling approaches, from retrospectively undersampled data. Results are shown using (a) the vessel-masked SSIM and (b) the error in the number of detected peaks. Black arrows indicate the increase in acceleration factor when using calib = 12 (at R = 15) instead of 32, which can be achieved without loss in image quality.

Supporting Information Figure S9: Comparison of image quality in reconstructions from retrospectively undersampled k-space data without calibration regions, using SAKE-calibration for sensitivity estimation, to image quality in reconstructions using 12$\boldsymbol{\times}$12 sampled lines in k-space for calibration. After sensitivity estimation, both approaches were reconstructed using the same compressed sensing pipeline. Results are shown as the mean SSIM ± the standard deviation across the 4 slabs shown in Figure 1.
